# FgLEU1 Is Involved in Leucine Biosynthesis, Sexual Reproduction, and Full Virulence in *Fusarium graminearum*

**DOI:** 10.3390/jof8101090

**Published:** 2022-10-17

**Authors:** Shaohua Sun, Mingyu Wang, Chunjie Liu, Yilin Tao, Tian Wang, Yuancun Liang, Li Zhang, Jinfeng Yu

**Affiliations:** Key Laboratory of Agricultural Microbiology, College of Plant Protection, Shandong Agricultural University, Tai’an 271018, China

**Keywords:** *Fusarium graminearum*, isopropylmalate isomerase, branched chain amino acid, FgLEU, virulence

## Abstract

Fusarium head blight (FHB) caused by *Fusarium graminearum* is a significant disease among cereal crops. In *F. graminearum*, biosynthesis of leucine, which is a branched chain amino acid, is achieved by converting α-isopropylmalate to β-isopropylmalate catalyzed by isopropylmalate isomerase encoded by *LEU1*. Considering the potential for targeting this pathway by fungicides, we characterized the gene *FgLEU1* (FGSG-09589) in the *Fusarium graminearum* genome using bioinformatics methods. For functional characterization, we constructed a deletion mutant of *FgLEU1* (Δ*LEU1*) through homologous recombination. Compared with the wild-type strain PH-1, Δ*LEU1* showed slower colony growth and fewer aerial mycelia. Leucine addition was needed to ensure proper mutant growth. Further, Δ*LEU1* showed decreased conidial production and germination rates, and could not produce ascospores. Moreover, Δ*LEU1* showed complete loss of pathogenicity and reduced ability to produce deoxynivalenol (DON) and aurofusarin. Upstream and downstream genes of *FgLEU1* were significantly upregulated in Δ*LEU1*. Contrary to previous reports, the deletion mutant was more resistant to osmotic stress and cell wall-damaging agents than the wild-type. Taken together, FgLEU1 plays a crucial role in leucine synthesis, aerial mycelial growth, sexual and asexual reproduction, pathogenicity, virulence, and pigmentation in *Fusarium graminearum*, indicating its potential as a target for novel antifungal agents.

## 1. Introduction

Fusarium head blight (FHB) is a serious disease of wheat, barley, and other small grains, usually resulting in lower grain yields, lower quality, and mycotoxin contamination [1]. *Fusarium graminearum* is recognized as the pathogen of FHB worldwide [1,2]. Ascospores of *F. graminearum* overwinter on crop residues such as maize, wheat, and barley, then infect the plants upon germination in the following spring and are spread by conidia during flowering [2]. FHB reduces grain quality through the destruction of starch and protein and by the accumulation of mycotoxins such as the trichothecene derivative, deoxynivalenol (DON); these changes can occur within a few weeks [3], and toxin pollution is inevitable once the seeds are infected [2]. DON is a threat to both human and animal health [4,5,6], and its synthesis in *F. graminearum* involves the Tri gene cluster [7]. Recently, some microorganisms have been found to degrade DON [8,9], but their use in agriculture is limited. The current control of FHB relies on some fungicides [2] and pesticides, which can cause great harm to plants and human health. Control of FHB and mycotoxin contamination thus remains challenging due to the lack of resistant wheat cultivars to FHB and limited effective fungicides against *F. graminearum* [1]. Considering this situation, the development of effective new fungicides that are less toxic and effective is an urgent need.

Three branch-chain amino acids (BCAAs), including isoleucine, leucine, and valine, cannot be synthesized by humans and other mammals, but can only be synthesized by bacteria, archaea, fungi, and plants [10]. Therefore, enzymes involved in the biosynthesis of BCAAs can be effective targets for fungicides. For example, sulfonylureas inhibit acetylhydroxyl synthase, the first enzyme in the branched chain amino acid synthesis pathway, and are thus used as feedstocks for fungicides [11] and herbicides [12,13,14]. Biosynthesis of branched amino acids is highly conserved in fungi, bacteria, and plants [14]. It involves a common pathway catalyzed by the same four enzymes [10] that leads to conversion of pyruvate and α-ketobutyrate to valine and isoleucine and a branch that leads to the conversion of α-ketoisovalerate, the immediate precursor of valine, to leucine [15]. Leucine synthesis begins with 3-methyl-2-oxobutanoate. The first step is the condensation of 3-methyl-2-oxobutanoate and acetyl-CoA catalyzed by isopropylmalate synthase (IPMS) to form 2-isopropylmalate. The second step is catalyzation by isopropylmalate isomerase (IPMI), also known as isopropylmalate dehydratase, and involves conversion of 2-isopropylmalate (2-IPM) to 3-isopropylmalate (3-IPM). In the third step, 4-methyl-2-oxopentanoate is synthesized by the oxidative decarboxylation of isopropyl malate dehydrogenase (IPMDH). The reversible reaction catalyzed isopropylmalate isomerase encoded by the *LEU1* gene is an indispensable step in leucine synthesis [10], similar to the reactions catalyzed by the aconitase family monomeric proteins, which contain [4Fe-4S] clusters [16].

BCAAs serve as building blocks for all life forms and act as important nutritional signals that mediate protein synthesis, glucose homeostasis, and nutrient-sensitive signaling pathways [14]. *LEU1* gene expression is highly conserved in different species, and mainly affects the leucine biosynthesis pathway. Leu1 acts as an isopropylmalate isomerase to isomerize α-isopropylmalic acid into β-isopropylmalic acid. The functions of *LEU1* homologs have been studied in many organisms. In *S. cerevisiae*, the expression product of the *LEU1* gene acts as an isopropylmalate isomerase and is regulated by Leu3p [15]. Mitochondrial Fe-S cluster (ISC) synthesis is regulated by the leucine biosynthetic pathway [17]; the role of Leu1 in iron metabolism has also been demonstrated in *Cryptococcus neoformans* [18]. In a subsequent study, LEU1 was suggested to combine with the regulator of the H^+^-ATPase in the vacuolar and endosomal membranes (REVA) to jointly regulate V-ATPase activity, which may affect the tolerance of *F. raminearum* to Ca^2+^ [19].

Several genes involved in leucine biosynthesis have also been studied in *Magnaporthe oryzae*, a model filamentous fungus. The results showed that deletion of leucine biosynthesis genes affected the development and pathogenicity of fungi [20,21]. According to Wei et al., leucine biosynthesis may be related to the degradation of glycogen and lipid droplets, which is related to the energy supply in fungi [21]. In *F.graminearum*, *FgLEU2A/B* are two paralogous downstream genes of *FgLEU1*; knockout of these genes was shown to inhibit the synthesis of 3-isopropylmalate dehydrogenase (IPMD), leading to severe damage to mycelial growth and pathogenicity [22]. These results indicate that inhibition of leucine synthesis affects the normal life activities of the fungus.

Considering the importance of leucine biosynthesis and its potential as a target for fungicides, we characterized the gene *FgLEU1* (FGSG-09589) in the *F.graminearum* genome in this study. We then utilized polyethylene glycol-mediated fungal transformation to yield a *FgLEU1*-deficient mutant strain and examined the functional role of *FgLEU1* in vegetative growth, asexual and sexual development, plant infection, and other traits of *F. graminearum*.

## 2. Materials and Methods

### 2.1. Fungal Strains and Culture Conditions

*F. graminearum* PH-1 was used as the wild-type (WT) strain for constructing the mutants (Δ*LEU1*) and complementary (Δ*LEU1*-C) strains. All the strains were cultured at 25 °C on potato dextrose agar medium (PDA), complete medium (CM), fructose gelatin agar (FGA), or yeast extract peptone dextrose (YEPD). For mycelial growth assays, all strains were grown at 25 °C on PDA medium and CM medium for 4 days. The colony diameter was measured using the crossed wire method. The average of the data obtained was processed. For the aerial hyphal growth assay, 5 mL of sterilized PDA medium was added to the test tube (1.5 cm in diameter). All strains were inoculated and grown for 5 days at 25 °C in test tubes. Hyphal quality was measured by filtration collection of solid materials after culture for 5 d in PDB, rinsed with sterilized water, dried in an oven at 65 °C, followed by determination of mycelial dry weight. All experiments were repeated three times. To induce asexual reproduction, mycelia were shaken in liquid carboxymethyl cellulose (CMC) medium at 180 rpm. Fungal mycelia were harvested from potato dextrose broth (PDB) and used for extracting total genomic DNA and RNA. *Escherichia coli* DH5a was used for normal bacterial transformation and was cultured at 37 °C. To test cell wall integrity, the colony diameter was measured using a CM plate with 0.4 mg/mL Congo Red (CR) added. Exogenous amino acids were added to the FGA at different concentrations, as indicated in the figures. Five 5 mm mycelial plugs of the wild-type strain and mutants were collected from the edge of a 3-day-old colony and incubated in the plates. Carrot agar was used to assess sexual reproduction. The experiments were repeated three times, with three replicates each.

### 2.2. Generation of FgLEU1 Deletion Mutants

We found the sequence of *FgLEU1* (FGSG_09589) from the *F. graminearum* database. To detect *FgLEU1*, we first PCR-amplified *FgLEU1*-A and *FgLEU1*-B using the primer set *FgLEU1*-A-F/ *FgLEU1*-A-R and *FgLEU1*-B-F/ *FgLEU1*-B-R (Table S1) and *F. graminearum* PH-1 genomic DNA as templates. The hygromycin B phosphotransferase (HPH) gene was amplified from plasmid pCB1003 with primer pairs HYG-F/HY-R and YG-F/HYGR (Table S1). The fusion cassette containing the gene-flanking sequences and the HPH gene was obtained by split-marker PCR, and the resultant PCR product was purified and stored at −20 °C for protoplast transformation PCR conditions: 200 mM primers, 200 mM dNTP, 1 unit of Phanta Max Super-Fidelity DNA Polymerase (Vazyme Biotech Co, Jiangsu, China, CAT: P505-d1), and the PCR fragments were transformed into the protoplasts of PH-1 using PEG to generate Δ*FgLEU1* mutants. TB3 medium supplemented with hygromycin B (300 mg/L) was used for preliminary screening of the transformations. Single spore isolation and PCR identification were performed. Southern blotting analyses of *FgLEU1* deletion mutants were performed using DIG High Prime DNA Labeling and Detection Starter Kit I, according to the manufacturer’s instructions (Roche Diagnostics, Mannheim, Germany). The probes were PCR-amplified using the primer pair t*LEU1*F/R (Table S1). The strains were stored in sterile water at −20 °C for further experiments.

### 2.3. Gene Complementation

To prepare a complementation strain, a 4.5 kb DNA fragment containing the full-length *FgLEU1* sequence, and its promoter sequence was amplified by PCR with the primer pair FgLEU1-CF/R. Then, the recombinant plasmid pYF11 using the yeast in vivo recombination approach (Roche Diagnostics, Mannheim, Germany), obtain recombinant plasmid pYF11-*FgLEU1*. The recombinant plasmid was transformed into protoplasts of the Δ*LEU1* mutants to produce Δ*LEU1* (Δ*LEU1*-C) complemented strains, which were selected using 200 mg/mL G418 and were identified by PCR using the primer pair Δ*LEU1*-CF/R (Table S1).

### 2.4. Asexual and Sexual Reproduction Assays

For conidiation assays, two 5 mm mycelial plugs of each strain were placed in 30 mL CMC medium (1 g KH_2_PO_4_, 2 g NaNO_3_, 1 g yeast extract, 0.5 g MgSO_4_·7H_2_O, and 15 g sodium carboxymethyl cellulose in 1000 mL distilled H_2_O) for 3 days in a shaker at 180 rpm at 25 °C. The number of conidia was counted for each strain using a hemocytometer. To determine the germination rate of conidia, 5-day-old conidia were resuspended in yeast extract peptone dextrose (YEPD) medium (yeast extract, 10.0 g; peptone, 20.0 g; glucose, 20.0 g; distilled water, 1000 mL), in a final volume of 80 mL, then shaken at 180 rpm, 25 °C for 4–6 h, followed by examination under a fluorescence microscope (Eclipse 90i, Nikon, Tokyo, Japan). Each experiment was performed in triplicate.

For sexual reproduction assays, each strain was inoculated on carrot medium for 7 days, aerial hyphae were pressed down with 1 mL of sterile 2.5% Tween 60 solution for sexual reproduction assays and cultured for 21 days at 25 °C under black light. Perithecium formation was examined using a microscope (Leica MZ10 F, Germany). Each experiment was repeated three times.

### 2.5. RNA Extraction and Quantitative Real-Time PCR (qRT-PCR)

To determine the expression levels of the genes to be determined, the same amounts of conidia of strains were inoculated into PDA and glucose yeast extract peptone (GYEP) medium and cultured in a shaker at 25 °C and 180 rpm for 3 days. The total RNA of wild-type PH-1 and deletion mutants was isolated from the hyphae using TransZol Up (TransGen Biotech, Beijing, China). qRT-PCR experiments were performed according to the manufacturer’s instructions. The 2^−ΔΔCT^ method was used to quantify the transcription levels relative to the glyceraldehyde-3-phosphate dehydrogenase (*GAPDH*) gene as a reference. The primers used are shown in Table S1.

### 2.6. Plant Infection and DON Production Assays

To assess the virulence of *F. graminearum* on wheat heads, conidia were harvested from 5-day-old CMC cultures and resuspended in sterile distilled water at 4 × 10^5^ spores/mL [23]. Briefly, a 10 μL conidial suspension was injected into the middle lower spikelet of wheat heads from susceptible wheat cv. Jimai 22 during flowering [24], and the inoculated wheat heads were sprayed with sterile distilled water and then wrapped in small plastic bags for 48 h to maintain high humidity. The wheat heads were then photographed and assayed 14 days after inoculation [25]. To assess the virulence of *F. graminearum* on corn silk, a hyphal plug (5 mm in diameter) was inoculated on four fresh corn silks placed on plates at 25 °C and high humidity. The results were observed at 5 dpi, and each experiment was performed in triplicate. To determine DON production and ergosterol levels. DON toxin used ergosterol as internal reference, and 25 g of autoclaved healthy wheat kernels were inoculated with eight mycelial plugs of each strain for 21 days at 25 °C. DON samples were quantified by liquid chromatography and tandem mass spectrometry, as described previously [26]. Each experiment was performed in triplicate.

### 2.7. Statistical Analysis

DPS data processing system was used for data analysis. Data were presented as the mean ± standard error values, and the differences among variables were analyzed using Duncan’s multiple range test with *p* < 0.05.

## 3. Results

### 3.1. Identification of FgLEU1 in F. graminearum

**Figure 1:**
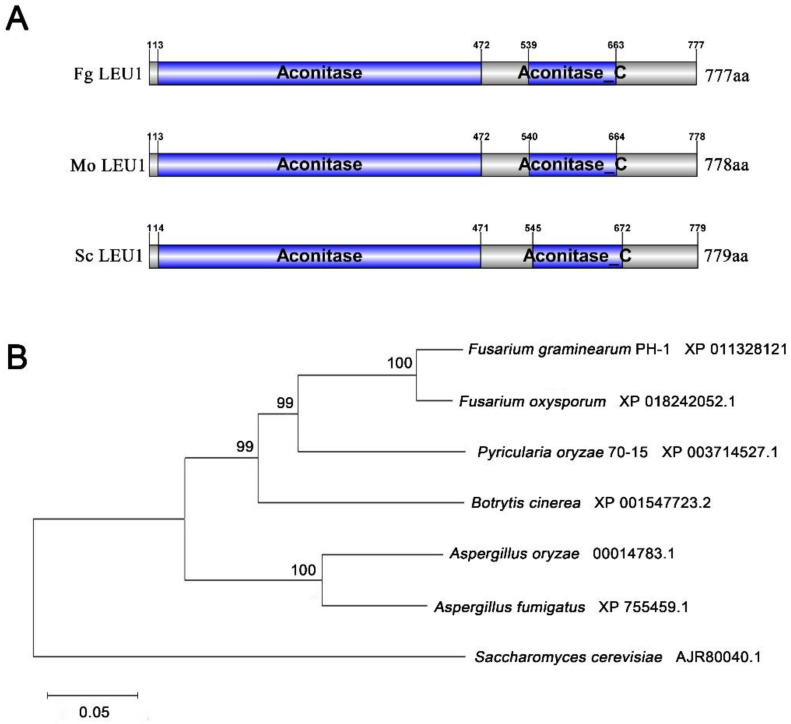
Sequence and phylogenetic analysis of LEU1. (**A**) Sequence and phylogenetic analysis of LEU1 in *Fusarium graminearum* (Fg), *Magnaporthe oryzae* (Mo), and *Saccharomyces cerevisiae* (Sc). Aconitase: An enzyme that catalyzes the stereo-specific isomerization of citrate to isocitrate via cis-aconitate in the tricarboxylic acid cycle through a non-redox-active process. Aconitase C: The Aconitase C-terminal domain, which undergoes conformational change in the enzyme mechanism. (**B**) A phylogenetic tree of LEU1 with homologues from other fungal species was constructed using the neighbor-joining method in MEGA version 7.0. Numbers at the nodes in the rooted tree represent the bootstrap value after 1000 replications. GenBank accession numbers are presented after the fungal species names. The bar indicates 0.05 distance units.

One putative LEU1 (FGSG_09589, designated as FgLEU1) in *F. graminearum* was retrieved by a BLAST search in National Center for Biotechnology Information (NCBI Blast: https://blast.ncbi.nlm.nih.gov/Blast.cgi) with *M. oryzae* LEU1 (MGG_01553) as the query. The *FgLEU1* gene is predicted to encode a 777-amino acid protein showing 62.47% identity with *S. cerevisiae* and 63.42% identity with *M. oryzae*. SMART-PFAM analysis revealed that FgLEU1 contains two different regions: aconitate hydratase and an Aconitase C-terminal domain. Phylogenetic analysis using the neighbor-joining method indicated that LEU1 is highly conserved among *F. graminearum* and other fungi (Figure 1).

To determine the biological function of FgLEU1 in *F. graminearum*, gene replacement constructs were generated and transformed into the wild-type (WT) strain PH-1 to generate *FgLEU1* deletion mutants (Δ*LEU1*), using homologous recombination; molecular verification of *FgLEU1* is shown in Figure S1. We obtained at least three independent deletion mutants for *FgLEU1* and further verified them by Southern blot analysis (Supplementary Figure S1). pYF11-*LEU1* complementation constructs were generated using the primer pairs CF/CR, and were transformed into the Δ*LEU1* mutant protoplasts to produce the complementation strain, Δ*LEU1*-C. The expression levels of *FgLEU1* were assessed using qRT-PCR analysis (Supplementary Figure S1). Δ*LEU1* mutants did not express the corresponding target genes, whereas the expression levels of *FgLEU1* were restored in the complementation strains.

### 3.2. FgLEU1 Is Responsible for Hyphal Growth, Conidiation, and Conidial Germination

**Figure 2:**
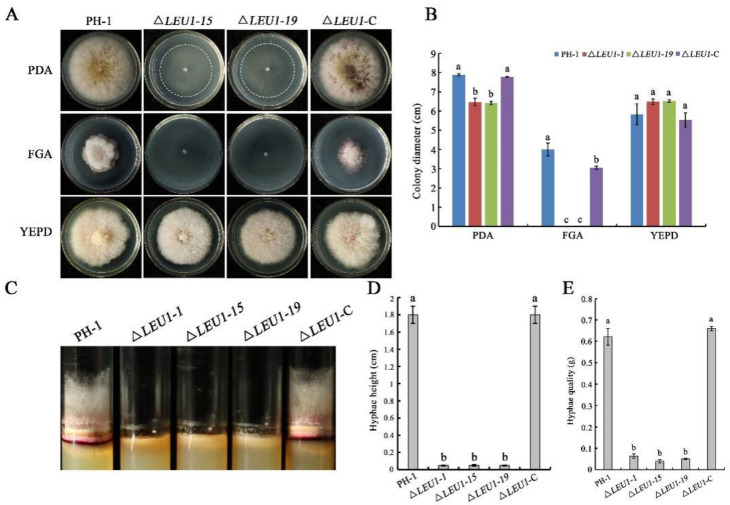
Hyphal growth of PH-1, Δ*LEU1*, and Δ*LEU1*-C strains. (**A**) Colonies of the wild type (PH-1), *FgLEU1* deletion mutants, and complemented strains were cultured on PDA, FGA, and YEPD agar plates after 4 days at 25 °C. (**B**) Height of aerial mycelium in test tubes containing PDA medium after 4 days at 25 °C. (**C**) Colony diameters of strains cultured on PDA, FGA, and YEPD plates. (**D**) Colony height of strains cultured in tubes with PDA. (**E**) Hyphal quality of strains cultured in potato dextrose broth (PDB) at 25 °C for 5 days at 180 rpm. Different letters on the bars for each treatment indicate significant differences at *p* < 0.05 by Duncan’s multiple range test. Measurements represent the average of three independent experiments. All experiments were repeated three times with three replicates at each time.

To determine the role of *FgLEU1* in hyphal growth, Δ*LEU1* mutants were inoculated onto PDA, fructose gelatin agar (FGA) and yeast extract peptone dextrose (YEPD) plates for growth assays. Compared with the wild-type strain PH-1, on PDA, the colony diameter of the Δ*LEU1* mutant was reduced by approximately 18.4%, hyphal quality was reduced by approximately 91.6% (Figure 2A,B), and aerial hyphae in germ tubes were significantly shorter than those in PH-1(Figure 2C,D). Δ*LEU1* mutants could not grow on the nutritionally deficient FGA plate but grew normally on YEPD medium with sufficient nutrition.

**Figure 3:**
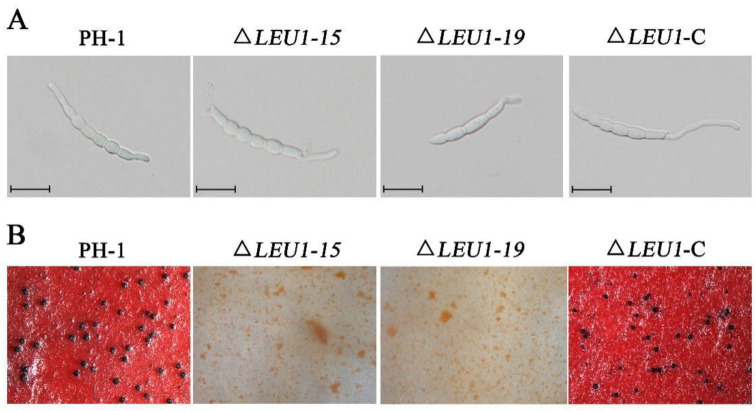
Conidial germination and sexual reproduction of *FgLEU1* deletion mutants. (**A**) All strains were incubated in liquid YEPD for 4 h at 25 °C with 180 rpm and examined for germination. Scale bar = 20 μm. (**B**) Sexual development of the strains. Perithecia were visualized as black structures on carrot agar plates. Photographs were taken at 21 days post self-fertilization. All experiments were repeated three times with three replicates at each time.

Compared to the WT strain, conidiation in 5-day-old cultures of Δ*LEU1* mutants in carboxymethyl cellulose (CMC) medium was reduced by approximately 78.9% (Table 1), but their morphology did not change significantly. When incubated on yeast extract peptone dextrose (YEPD) medium, approximately 60.34% of the PH-1 conidia germinated at 4 h post-incubation (hpi). Under the same conditions, the germination rate of Δ*LEU1* conidia was 42.32%, and the length of the germ tubes was slightly shorter than that in PH-1 (Figure 3A). Taken together, these results indicated that the presence of FgLEU1 is required for cells for sexual reproduction.

### 3.3. FgLEU1 Is Necessary for Sexual Reproduction

Sexual reproduction plays a critical role in the infection cycle of *F. graminearum* [27]. Therefore, we assessed the sexual reproduction of the mutants and wild type (PH-1) on carrot agar plates at 2–3 weeks post-fertilization. PH-1 and Δ*LEU1*-C produced abundant and normal perithecia, whereas the mutants did not produce perithecia (Figure 3B). These results indicate that *FgLEU1* plays a crucial role in sexual reproduction and thus affects the disease cycle seriously.

### 3.4. FgLEU1 Is Essential for Pathogenicity and DON Production

**Figure 4:**
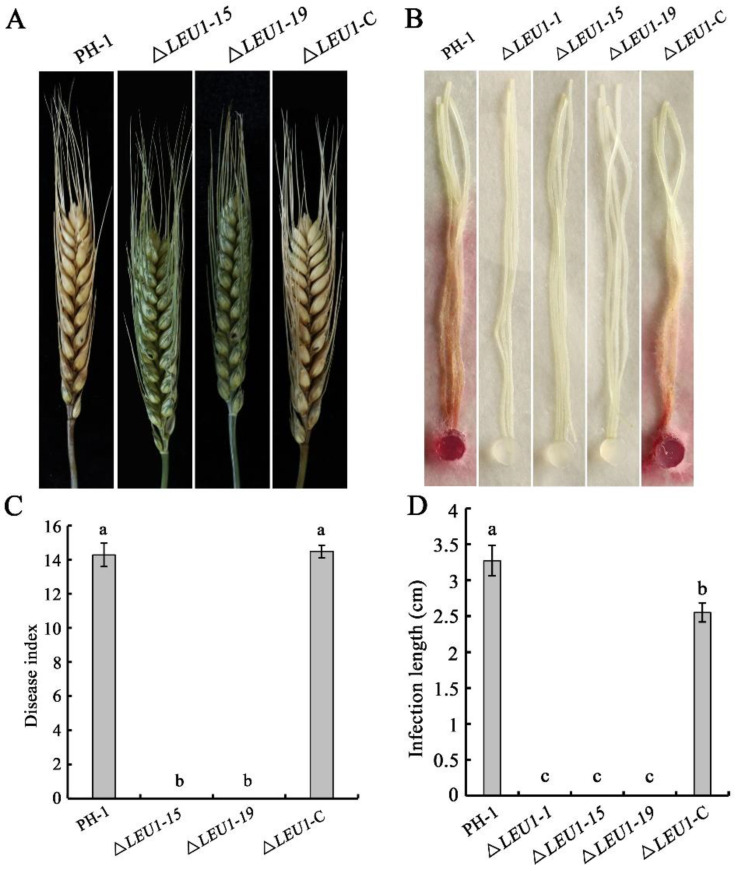
Virulence of the WT strain and *FgLEU1* deletion mutants on wheat heads and corn silks. (**A**) Disease symptom on flowering wheat heads caused by PH-1, ∆*LEU1*, and ∆*LEU1*-C strains after injection with conidial suspension (10 μL, 4 × 10^5^ conidia/mL). Images were taken at 14 days post-inoculation (dpi). (**B**) Brown necrosis caused by PH-1, ∆*LEU1*, and ∆*LEU1*-C strain mycelial plugs on corn silks. Images were taken at 5 dpi at 25 °C. (**C**) Disease indices of *F. graminearum* determined at 14 dpi. At least 20 wheat heads were examined in each replicate. Error bars represent the standard errors of the means. Different letters on the bars for each treatment indicate significant differences at *p* < 0.05 by Duncan’s multiple range test. (**D**) The length of brown necrotic tissue upon infection was determined at 5 dpi. At least 20 corn silks were examined in each replicate. Different letters on the bars for each treatment indicate significant differences at *p* < 0.05 by Duncan’s multiple range test.

To determine the role of *FgLEU1* in pathogenicity, pathogenicity assays were conducted to determine the effect of ∆*LEU1* on flowering wheat heads and corn silks. On wheat heads inoculated with 4.0 × 10^5^ conidia/mL either PH-1 or Δ*LEU1*-C, typical scab symptoms spread from the inoculated to uninoculated spikelets within 14 days post inoculation (dpi). To our surprise, the mutant strain was completely unable to infect the spikelet, and the disease index score was 0 (Figure 4A,C). This was also confirmed by the results of corn silk infection assays; PH-1 and Δ*LEU1*-C could form typical brown lesions at 5 dpi, but the mutants could not (Figure 4B,D). These results indicate that *FgLEU1* plays an important role in pathogenicity.

To determine the effect of *FgLEU1* on DON production, we assayed DON production in toxin-producing rice medium inoculated with all strains for 21 days. The results showed that the ∆*LEU1* mutant produced significantly less DON than PH-1 and ∆*LEU1*-C strains (Table 1). To confirm this finding, the expression of *Tri5*, *Tri6*, and *Tri10* (trichothecene biosynthesis), which are essential for DON synthesis, was analyzed by quantitative reverse transcription-polymerase chain reaction (qRT-PCR). As shown in Table 2, the expression of *Tri* genes was significantly downregulated in Δ*LEU1* mutants compared to those in the WT strain. As expected, supplementation with leucine alleviated this change. These results indicate that *FgLEU1* is involved in regulating *Tri* expression and DON synthesis. Based on these results, we concluded that leucine is involved in the DON synthesis pathway.

### 3.5. Deletion of FgLEU1 Results in Reduced Red Pigmentation

**Figure 5:**
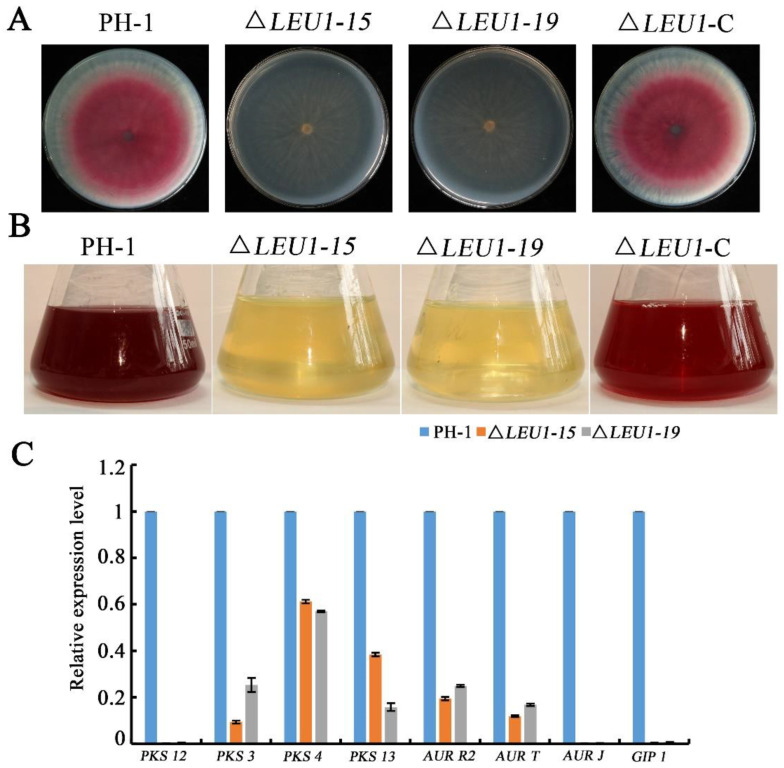
Pigment production and relative expression level of aurofusarin biosynthesis genes by the wild-type strain PH-1 and *FgLEU1* deletion mutants. (**A**) Red pigment aurofusarin formation by the wild-type PH-1, *FgLEU1* deletion mutants, and complementation strains cultured on PDA at 25 °C for 4 days. (**B**) Red pigment aurofusarin formation by the wild-type PH-1, *FgLEU1* deletion mutants, and complementation strains cultured in liquid medium in flasks containing 50 mL of PDB at 180 rpm, 25 °C for 5 days. (**C**) Relative expression levels of aurofusarin biosynthesis-related genes. *GAPDH* was used as an internal control. Gene expression in PH-1 was set to 1.0 (*p* < 0.05).

Deletion of *FgLEU1* also led to reduced accumulation of red pigment; PH-1 and ∆*LEU1*-C formed pink colonies at 3 dpi, whereas ∆*LEU1* formed slightly pale yellowish colonies on the bottom of the plates (Figure 5A). In PDB flasks, the filtrate of the wild-type and complemented strains appeared red, whereas the color of the mutant strain was almost unchanged (Figure 5B). These results indicated that FgLEU1 is involved in the production of the red pigment, aurofusarin.

To further confirm this conclusion, we performed qRT-PCR to determine the expression levels of *PKS12*, *PKS3*, *PKS4*, *PKS13*, *AURT*, *AURJ*, *AURR2*, and *GIP1* genes related to the biosynthesis of aurofusarin. As shown in Figure 5C, deletion of the *FgLEU1* gene led to the downregulation of these genes, thus confirming that *FgLEU1* deletion affects pigment biosynthesis.

### 3.6. FgLEU1 Plays a Crucial Role in Leu Biosynthesis in F. graminearum

**Figure 6:**
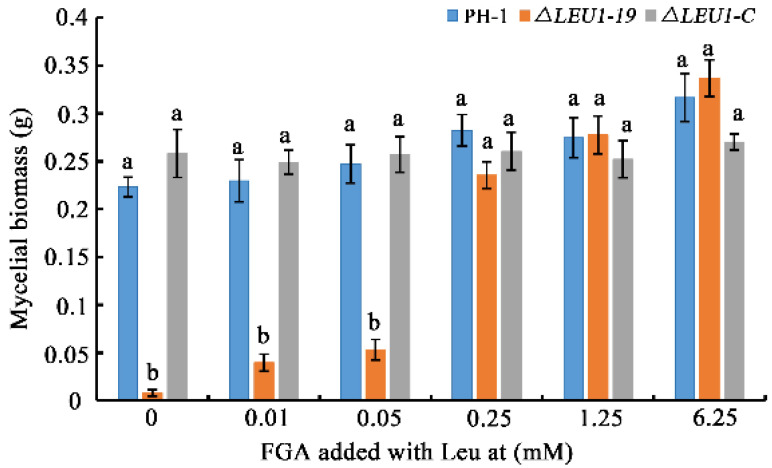
The mycelial biomass of wild-type, Δ*LEU1*, and ∆*LEU1*-C strains with different concentrations of leucine. Different concentrations of leucine (final concentrations 0, 0.01, 0.05, 0.25, 1.25, and 6.25 mM) were added to fructose gelatin broth medium and the strains were cultured at 25 °C, 180 rpm, for 6 days. Mycelium was collected from the medium, dried at 65 °C and weighed after two days. Error bars represent the standard errors of the means. Different letters on the bars for each treatment indicate significant differences at *p* < 0.05 by Duncan’s multiple range test.

As shown in Figure 2, *FgLEU1* deletion resulted in amino acid auxotrophy in *F*. *graminearum*. ∆*LEU1* could not survive normally on PDA, a semi-synthetic medium. It could not survive in the absence of amino acids on FGA. In contrast, the auxotrophy of Δ*LEU1* could be completely recovered when cultured on the full-nutrition medium, YEPD. Therefore, we examined whether the growth defects caused by nutrient deficiency could be repaired by exogenous leucine added to fructose gelatin broth (FGB) medium. The results of mycelial biomass analysis showed that the nutritional deficiency of mutants could be partially recovered by exogenous leucine addition. When the terminal leucine concentration was greater than 0.25 mM, the growth defects of the mutants were almost completely eliminated (Figure 6).

### 3.7. Effects of FgLEU1 Deletion on the Maintenance of Cell Wall Integrity

**Figure 7:**
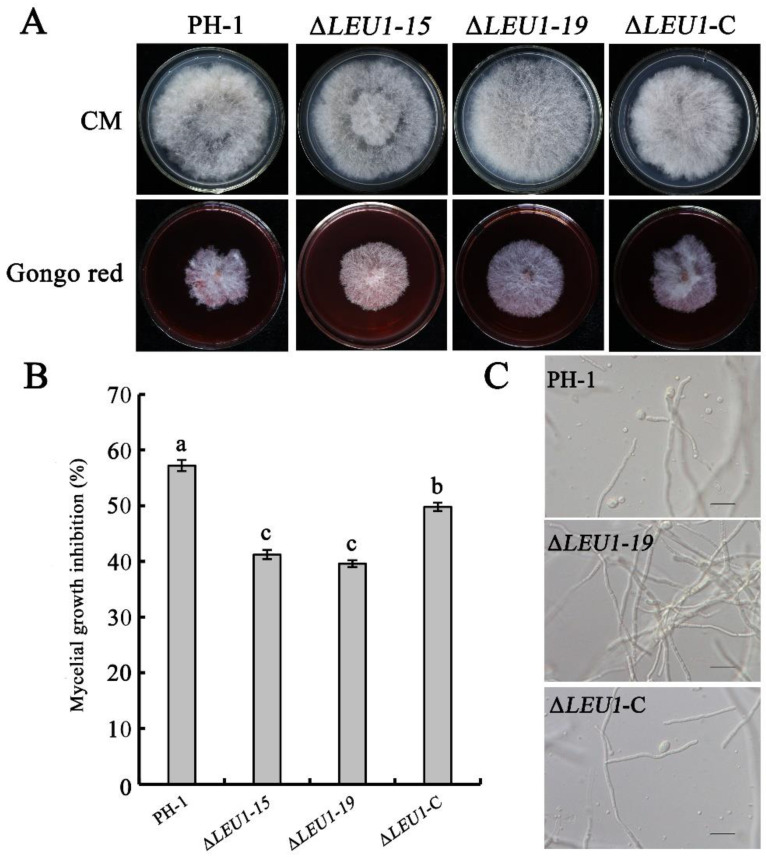
Role of *FgLEU1* in resistance to cell wall-damaging agents and cell wall-degrading enzymes. (**A**) Comparison of PH-1, Δ*LEU1*, and Δ*LEU1*-C after 4 days of incubation at 25 °C on CM plates with or without 300 mg/mL Congo Red (CR). The plates were photographed at 4 days post cultivation. (**B**) Mean rates of mycelial growth inhibition by CR on PH-1, Δ*LEU1*, or Δ*LEU1*-C strains at 4 days post cultivation. Different letters on the bars for each treatment indicate significant differences at *p* < 0.05 by Duncan’s multiple range test. Bar = 20 µm. Bars denote standard errors from three experiments. Values on bars followed by the same letter for each treatment indicate absence of a significant difference at *p* < 0.05. (**C**) After treatment with lysozyme, driselase, and snailase for 4 h at 30 °C, the mycelia of PH-1 and Δ*LEU1*-C mutants were well digested and numerous protoplasts were released, but this was not observed for Δ*LEU1*.

To investigate the possible role of *FgLEU1* in maintaining cell wall integrity (CWI), we assessed the mycelial growth in all strains in the presence of cell wall-damaging agents. Congo red (CR) specifically binds substances in the cell wall, blocks normal cell wall assembly, produces cell wall stress, and inhibits fungal growth [28]. All strains were incubated in CM medium supplemented with 300 μg/mL of CR. Δ*LEU1* showed increased tolerance to CR compared to PH-1 and ∆*LEU1*-C (Figure 7A,B). For further confirmation, all strains were treated with lysozyme, driselase, and snailase for 4 h at 30 °C. The mycelium of PH-1 was enzymatically cleaved and released many protoplasts, whereas most mycelia of Δ*LEU1* were not enzymatically cleaved and remained intact (Figure 7C). These results suggest that *FgLEU1* is associated with the maintenance of CWI.

## 4. Discussion

The physiological and metabolic processes of plant pathogenic fungi are receiving extensive attention for the selection of targets in fungicide development. Branched chain amino acids (BCAAs) include valine, leucine, and isoleucine, which can only be synthesized by bacteria, archaea, fungi, and plants; animals, including humans, can only obtain these from their diet [10,29]. It is thus promising to examine enzymes in the BCAA biosynthetic pathway as potential targets. Amino acid synthesis plays an important role in the growth and development of plant pathogenic fungi and in host plant infestation [30,31]. This has been confirmed in several previous studies on *F. graminearum*. For example, enzymes involved in the biosynthesis of arginine (Arg)(Amt1), leucine (Leu), isoleucine (Ile), valine (Val) (Ilv1, Ilv2, Ilv3, Ilv5, Ilv6), and glutathione (Oxp1, Oxp2), affect the growth and development of *F. graminearum* [14,32,33,34,35,36]. These results all indicate the link between amino acid malnutrition and morphological growth and the pathogenicity of phytopathogenic fungi. In this study, we demonstrated that targeting alternative leucine biosynthesis of FgLEU1 in *F. graminearum* leads to a significant reduction in the growth of mutant Δ*LEU1* strains, thereby inhibiting conidiogenesis. These results are consistent with previous findings.

LEU1 is dispensable for vegetative growth in *S. cerevisiae*, *M. oryzae,* and *C. neoformans* [18,20,37]. Based on our experimental results that Δ*LEU1* could not colonize the nutrient-deficient (nutrient-poor) FGA medium and that exogenous supplementation with Leu could effectively rescue the nutritional deficiency of the mutant, we concluded that leucine plays a conserved role in the morphogenesis of *F. graminearum*. Moreover, we found that 0.25 mM Leu is required for the normal growth of the strain.

The hyphae of *F. graminearum* form overwintering structures together with the host plant tissue, in the spring field, the airborne ascospores then become the main primary inoculum that infect the host flowering structures. The process of sexual development to produce ascospores plays a crucial role in the disease cycle [1,38]. The inability of the Δ*LEU1* mutant to produce perithecia and subsequent ascospores suggesting that its presence is required for cells for sexual reproduction in *F. graminearum*. Deletion of the *FgLEU1* gene leads to blockage of the disease transmission pathway, suggesting that FgLEU1 may be a useful fungicide target for controlling FHB.

Liu et al. [22] reported that LEU2 is involved in the pathogenicity of *F. graminearum*; MoLEU1, MoLEU2, and MoLEU4 are regulated by MoLEU3, which also affects the pathogenicity of *M. oryzae* [21]. Plant infection assays revealed that the Δ*LEU1* mutant had significantly reduced infestability in wheat heads and corn silk. Reduced pathogenicity is influenced by many factors. For example, we found that the number and germination rate of mutant conidia were reduced (Table 1) and mycelial growth was inhibited under low nutrient conditions. During the spore germination and infestation stages, the nutrients required by the pathogenic fungus were derived exclusively from the internal stores, and the fungus only carries out its own nutrient synthesis after infestation [30]. The lack of nutrients in plants does not allow the pathogenic fungal growth. However, synthesis of leucine in the mutant strain is blocked; thus, the pathogenic fungus cannot grow properly to complete the infestation. The study by Tang et al. [20] agrees with this view.

Both DON and pigment production are important virulence factors. DON is a secondary metabolite produced by *F*.*graminearum*, and previous studies have shown that DON production significantly contributes to the fungal infestation in wheat spikes [39,40].

This study showed that DON production was reduced in Δ*LEU1* mutants (Table 1), suggesting that deletion of FgLEU1 interfered with DON production in *F. graminearum*. Furthermore, qRT-PCR analysis confirmed that the expression levels of trichothecene synthase genes, *Tri5*, *Tri6*, and *Tri10* were reduced in the Δ*LEU1* mutant; exogenous addition of leucine increased their expression, but its effect was not significant (Table 2).

Pigment production is accompanied by the growth stage of *F. graminearum*. As pigment deposition in the cell wall causes mycelium color changes, color can also be an indicator of growth in growth studies [41]. Mycelial color changes were also observed in the mutant strains in this study, and significant downregulation of pigmentation genes was observed in the *FgLEU1* deficient strain. These results are consistent with the reduced pathogenicity of the mutant, indicating that the *FgLEU1* gene plays an important role in the growth of *F. graminearum*. Further, *FgLEU1* deletion mutants showed reduced sensitivity to cell wall disruptors, which is consistent with the high tolerance to cell wall-lysing enzymes. These results differ from previous reports on mutants of BCAA synthesis genes in filamentous fungi [14,18,22,35].

Que et al. [42] previously proposed the metabolic pathway of leucine in *M. oryzae*. Combined with the results of qRT-PCR in this study, we concluded that the expression of LEU3 is not regulated by genes such as LEU1, LEU2, and LEU4. Deletion of LEU1 can significantly upregulate LEU2, LEU4, and BAT2 genes, indicating that LEU1 can affect the expression of its upstream and downstream genes.

In summary, we identified and elucidated the biological functions of FgLEU1 in the phytopathogenic fungus, *F. graminearum*. The results showed that FgLEU1 is essential for aerial hyphal growth, conidiation, virulence, pigmentation, and full pathogenicity in *F. graminearum*. These findings provide further evidence indicating the importance of leucine biosynthesis in the development of filamentous phytopathogens.

## Figures and Tables

**Table 1:** Conidiation, conidial germination perithecia counts, and DON production in the PH-1, Δ*LEU1*, and Δ*LEU1*-C strains.

Strain	Conidiation (10^5^ conidia/mL) ^α^	Germination (%) ^β^	Perithecia Counts ^γ^	DON Production (ppm) ^δ^
PH-1	7.33 ± 0.74 ^a^	60.34 ± 0.02 ^b^	38.44 ± 2.2305 ^a^	14.3733 ± 0.6 ^b^
Δ*LEU1-15*	1.67 ± 0.4 ^b^	42.32 ± 0.03 ^c^	0 ^b^	0.0133 ± 0.01 ^c^
Δ*LEU1-19*	1.42 ± 0.48 ^b^	41.44 ± 0.03 ^c^	0 ^b^	0.0367 ± 0.03 ^c^
Δ*LEU1*-C	7.12 ± 0.98 ^a^	71.91 ± 0.00 ^a^	38.55 ± 1.0943 ^a^	17.0533 ± 0.34 ^a^

All experiments were repeated three times with three replicates each time. Different letters behind data for each treatment indicate significant difference at *p* < 0.05 by Duncan’s multiple range test. ^α^ Conidiation in 100 mL of carboxymethylcellulose (CMC) cultures was examined after incubation at 25 °C, 180 rpm for 3 days. ^β^ Conidial germination was assayed after 4 h of incubation at 25 °C, 180 rpm. ^γ^ The number of perithecium in the mating plates of the PH-1 and mutants on carrot agar plates. ^δ^ DON and ergosterol (Erg) production was analyzed by HPLC-MS/MS. Rice samples inoculated with mycelial plugs were cultured for 20 days at 25 °C.

**Table 2:** Relative expression level of *Tri* (trichothecene biosynthesis) genes in the PH-1, Δ*LEU1*, and mutants supplemented with leucine.

Strain	Relative Expression Level		
	*Tri5*	*Tri6*	*Tri10*
PH-1	1 ± 0.02 ^a^	1 ± 0.01 ^a^	1 ± 0.02 ^a^
Δ*LEU1-19*	0.115 ± 0.02 ^b^	0.1691 ± 0.01 ^d^	0.0936 ± 0.00 ^b^
Δ*LEU1-19* + Leu	0.944 ± 0.02 ^a^	0.5274 ± 0.03 ^c^	1.0341 ± 0.03 ^a^
Δ*LEU1*-C	1.0257 ± 0.14 ^a^	0.8332 ± 0.02 ^b^	0.9622 ± 0.04 ^a^

Expression levels of several genes at the level of transcription. The relative expression level of *GAPDH* gene was used as an internal control. The gene expression in PH-1 was set to 1.0. Different letters behind data for each treatment indicate significant difference at *p* < 0.05 by Duncan’s multiple range test.

## Data Availability

Not applicable.

## Supplementary Materials

The following supporting information can be downloaded at: https://www.mdpi.com/article/10.3390/jof8101090/s1, Figure S1: The *FgLEU1* gene deletion strategy and confirmation in *F. graminearum*; Figure S2. Sexual reproductive morphology of the wild-type and revertant strains. Table S1: Primers used in this study.
