# Supplementary material for: FgLEU1 Is Involved in Leucine Biosynthesis, Sexual Reproduction, and Full Virulence in Fusarium graminearum
Source: J Fungi (Basel). 2022 Oct 17;8(10):1090. doi: 10.3390/jof8101090 (PMC9604659; doi:10.3390/jof8101090)
Supplement: Supplementary file 1 [file jof-08-01090-s001.zip › jof-1932540-supplementary.pdf]

Journal of *Fungi*

MDPI

Supplementary Materials

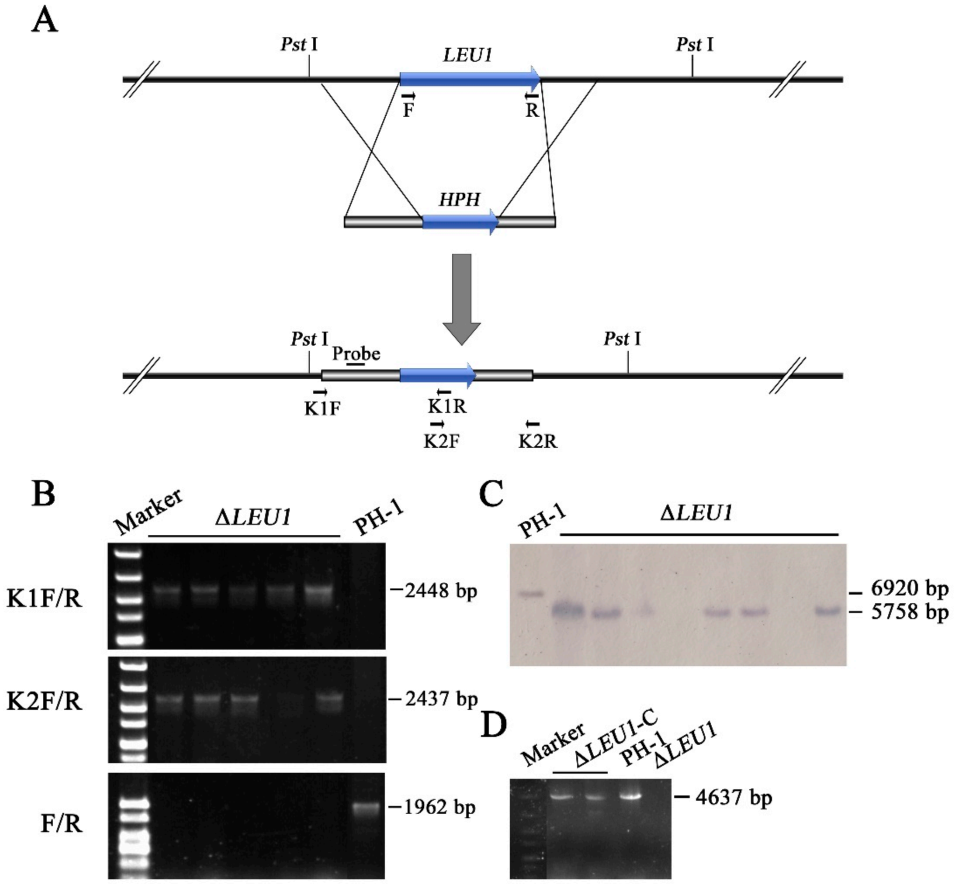

Figure S1. The *FgLEU1* gene deletion strategy and confirmation in *F. graminearum*. (**A**) Schematic diagram of the homologous recombination strategy using for constructing mutants. The Δ*LEU1* mutant was generated by substituting the *FgLEU1* gene fragment (2541 bp) with hygromycin phosphotransferase cassette (*HPH*, 1379 bp). The specific *Pst* I restriction enzyme cleavage sites were flagged on the upstream and downstream flanking regions, respectively. The deletion cassette containing the upstream and downstream flanking sequences was ligated and amplified with *hph* and transformed into the wild-type strain PH-1. Candidate mutants were filtered using identification primers, which are marked by arrows in the figure. (**B**) PCR analyses showed that the Δ*LEU1* mutants were correctly constructed. (**C**) Southern blot analysis of Δ*LEU1*. Southern blot hybridization analysis of PH-1 and Δ*LEU1* using a 309 bp *FgLEU1* upstream fragment as a probe. The genomic DNA preparation of each strain was digested using *Pst* I. The lengths of the fragments obtained from wild-type and mutant strains were 6920 bp and 5758 bp, respectively. (**D**) The mutant strain was transferred to the constructed plasmid with the target gene *FgLEU1* to build the reverting strain, and was verified by PCR.

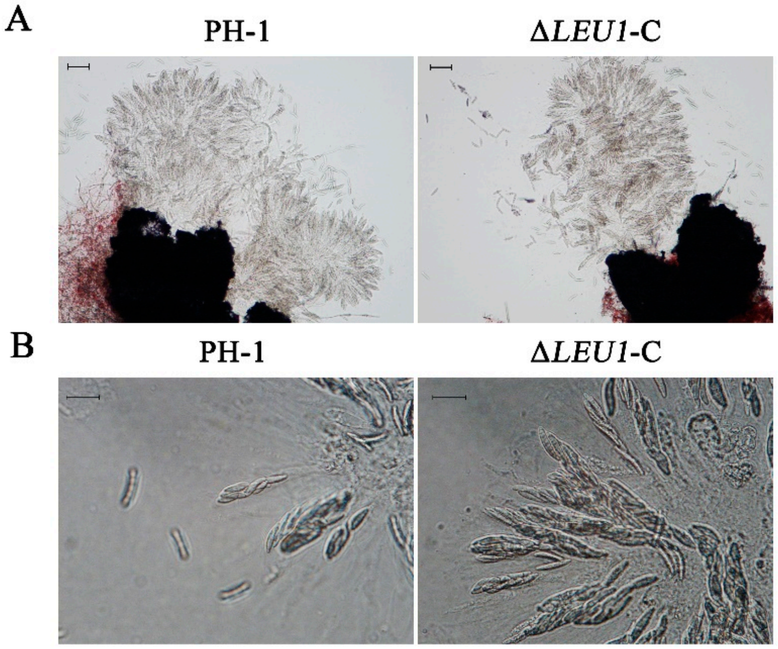

Figure S2. Sexual reproductive morphology of the wild-type and revertant strains. (**A**) Microscopic view of the perithecium in the wild-type and revertant strains. Both wild-type strains and revertant strains produced a normal perithecium. Scale bar = 50 μm. (**B**) Normal morphology of the ascus and ascospores in the perithecium of wild type strains and revertant strains. Scale bar = 20 μm.

Table S1. Primers used in this study.

Primer	Sequence (5′-3′)	Application
AF	GATATCAGACAGTCTTCAAAGGC	amplify *FgLEU1* 5′ flank sequence
AR	TTGACCTCCACTAGCTCCAGCCAAGCCTTTGACAATGA-GACCGCACTG	
BF	GAAATGAGTAGATGCCGACCGCGGTTTCTGGATA-CAACAAAATGGG	amplify *FgLEU1* 3′ flank sequence
BR	GGCTCTCATCTCCTTCAAACCCTAT	
HYG-F	GGCTTGGCTGGAGCTAGTGGAGGTCAA	amplify *HPH* N-terminal sequence
HY-R	GTATTGACCGATTCCTTGCGGTCCGAA	
YG-F	GATGTAGGAGGGCGTGGATATGTCCT	amplify *HPH* C-terminal sequence
HYG-R	AACCCGCGGTCGGCATCTACTCTATTC	
MF	CTCCTCAGACTCTTTACGACAAGGT	for identification of *FgLEU1* deletion transformants
MR	CAGATCCATCCTCCTTGTATCGAAG	
K1F	CGCAAGCAGGAATGTCGGTAAC	
K1R	TCGTCCATCACAGTTTGCCAGT	
K2F	TGGTCAGATCAGCCCACTTGT	
K2R	TTCACCATTCATCAGTCTCCCAC	
T F	ACGTGGCTTTTGGGGTTGTAC	amplify *FgLEU1* probe sequence
T R	TATCTTCCCGCATGTCTACCG	
C F	ACTCACTATAGGGCGAATTGGGTACTCAAATTGGTTCG-GAACAGGACATTCTTCTGG	*FgLEU1* complementation
C R	CACCACCCCGGTGAACAGCTCCTCGCCCTTGCTCAC-GCAGTGACGAAGTTTTCGAGTCAT	
GAPDH-F	CTTACTGCCTCCACCAACTG	qRT-PCR analysis
GAPDH-R	TGACGTTGGAAGGAGCGAAG	

Tri5-F	GAGTGTTTCATGCATGGCTACGTC
Tri5-R	CTGAGCCTCCTTCACATCGTCC
Tri6-F	CTGAGGGCATTCTGAGTAGCGACA
Tri6-R	CGTTATGTTTATCGGCACTTTG
Tri10-F	GCGACAGGAGCAAGAACATAA
Tri10-R	GGCGGCGTAAATCTGAGTG
PKS 12 F	ACGGCTTGCTCCTCCAGTCTC
PKS 12 R	CCAGCGAAGTTGTCAGGGTTAGTC
PKS 3-F	GAATGTCCTCCACGCCACCAAG
PKS 3-R	ACTCGCACAATGTCACTCGTAACC
PKS 4-F	TTGTTGACGACGAGCGAGTATTGG
PKS4-R	ACCAGTGCCATTGATGCCATGAC
PKS13-F	AGGCTGCTGCGGTAATTGTTCTC
PKS13-R	ATGGCTCTGACGGCTAGGGTTC
AUR T-F	GCCCATCTGGTTCCAAGGTATTCG
AUR T-R	CAGCCGACGCCGTTGATGAC
AUR J-F	GCCGCTGCTTATCCACACCTC
AUR J-R	GCCAATCACATGCTTGCTTACTGC
AUR R2-F	ATCGCCTTCTCACTCACGCAATG
AUR R2-R	CGCTGATCTCCACTGACAATGACC
GIP 1-F	CCGACGATTCTCTGCGACGAAG
GIP 1-R	CATCTGACCAAGGTGTGCCCATC
LEU 1-F	ATTGTTCTCCATGCCATCGGTCAG
LEU 1-R	CCATGCTCAGGCTGCGGATAAC
LEU 2-F	GACCTTGCTTCTTCGCCTCTGAC
LEU 2-R	GCCAAAGTAGATTCCGCCAGTGAG
LEU 3-F	CAAGCGTGGCGGCAATTACAAC
LEU 3-R	GGCGAGGATTGAGGAATGGTAGC
LEU 4-F	CGAGGCAACGGTCCCATTTCC
LEU 4-R	CGATGGCGTGCTCCTTGTAGTC
BAT 2-F	GCTTGCTGAGGCTGCGGATG
BAT 2-R	AGATGGATCGGACAGGGCTGAC
